# Documentation of Drug and Non‐Drug Allergies in Public Hospital Electronic Health Records: A National Survey

**DOI:** 10.1111/cea.70186

**Published:** 2025-12-02

**Authors:** Melvin Lee Qiyu, Erika Harnik, Claudia Gore

**Affiliations:** ^1^ Section of Inflammation, Repair and Development, National Heart and Lung Institute, Imperial College London London UK; ^2^ Department of Paediatric Allergy and Respiratory Medicine University Hospital Southampton NHS Foundation Trust Southampton UK; ^3^ Department of Paediatric Allergy Imperial College Healthcare NHS Trust, St. Mary's Hospital London UK

## Abstract

**Background:**

Inconsistent documentation of known allergies in electronic patient records (EPRs) poses a major patient safety risk. Unlike drug allergies, food and non‐drug allergens lack standardised documentation frameworks. This study evaluated how such allergies are recorded across NHS Trusts and the extent of avoidable harm from exposures.

**Methods:**

We conducted a national cross‐sectional, study. Freedom of Information (FOI) requests were sent to all 209 public (NHS) hospital Trusts (December 2024) to collect data on allergy‐related incidents, EPR availability, training and governance. An online survey was distributed to British Society for Allergy & Clinical Immunology (BSACI) members (February 2025) to capture clinician experiences, challenges and suggestions. Quantitative data were analysed using non‐parametric statistics; qualitative data were analysed using thematic analysis.

**Results:**

Responses were received from 194 Trusts (93%), with 145 providing complete datasets (582 hospitals). Sixty‐one different EPR platforms were identified, with 44 Trusts operating multiple systems. Across a median 10‐year span, 12,385 allergy‐related incidents were reported: 7724 (62%) drugs and 1277 (10%) food/non‐drug allergens. Over half of Trusts (54%) lacked a dedicated incident‐reporting category for food/non‐drug allergens, suggesting underestimation. Trusts without EPRs reported 140% more incidents than those with EPRs (*p* = 0.002). Those with in‐house allergy services, training and guidance recorded higher incident rates, consistent with enhanced recognition and reporting. The BSACI survey revealed challenges including poor EPR usability, limited coding options, weak alert systems and reliance on free text. Ninety percent of respondents supported national guidance on allergy documentation.

**Conclusion:**

Marked heterogeneity exists in NHS EPR systems and food/non‐drug allergy documentation practices. Trusts without EPRs experience higher reporting rates, while training and specialist services are associated with improved recognition. The absence of dedicated categories for food and non‐drug allergies contributes to systematic underreporting. These findings demonstrate how fragmented systems and inconsistent governance directly compromise allergy safety across UK hospitals.

## Key Messages

- 1277/12,385 (10%) allergy incidents related to administration of food/non‐drug allergens.
- Food and non‐drug allergen documentation on EPRs is suboptimal, secondary to training and EPR inconsistencies.
- 61 different NHS EPR‐systems; more allergy incidents occurred when no EPR or multiple EPRs used.

## Graphical abstract

These surveys reveal inconsistent electronic patient record (EPR) systems and poor documentation standards for non‐drug allergies, leading to high incident rates and misclassification. Standardised documentation, improved training and user‐friendly EPR platforms are urgently needed to enhance patient safety and reduce allergy‐related incidents across the NHS.

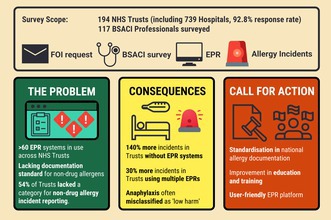

## 1 Introduction

Approximately 5%–10% of the general population report a drug allergy, most commonly to penicillin [1]. However, over 90% of these labels are inaccurate when formally assessed [2]. In contrast, food allergies affect fewer individuals but are increasing, especially in children. As of 2018, food allergies affected 4.0% of children under 5, 2.4% of school‐aged children, 1.7% of adolescents and 0.7% of adults—nearly triple the rates in 2008 [3]. Despite lower prevalence, food allergies account for a higher proportion of fatal anaphylaxis, particularly in children, often involving peanuts, tree nuts and cow's milk [4].

Although food allergies can cause life‐threatening reactions [5], documentation in electronic patient records (EPRs) remains inconsistent. Unlike drug allergies, which benefit from national guidance such as the NICE quality standard [6], food and non‐drug allergies lack structured documentation frameworks. This contributes to variable recording, clinical mismanagement and avoidable harm.

Hospital foodservice systems add further complexity, with limited communication between catering and clinical teams [7]. In an Australian study, 69 of 906 therapeutic meals were inaccurate, with food allergies and intolerances accounting for the highest proportion of errors (17%) [8].

While 85.3% of hospital anaphylaxis is drug‐related, food‐induced anaphylaxis remains significant, occurring at 1.5 per 5000 admissions [9]. Poorly structured allergy records—often vague or inconsistent—further compromise patient safety [10, 11].

Beyond food and drugs, several non‐drug substances can provoke allergic reactions, from contact dermatitis to anaphylaxis. A study from two U.S. academic medical centres identified diagnostic contrast agents and blood products as the most frequent non‐drug allergens [12]. Others include latex, chlorhexidine, medical adhesives, iodinated contrast media and surgical dyes such as patent blue [13, 14, 15, 16, 17, 18, 19]. Iodinated contrast agents cause reactions in 0.5%–3% of cases, with severe anaphylaxis in up to 0.2% [15].

A U.S. analysis of 744 allergy‐related clinical incidents underscored several contributory factors: incomplete or inaccurate documentation (50.4%), human error (23.5%), limitations in alert systems (17.1%), data exchange failures (12.4%) and inadequate EPR default settings (4.0%) [13]. These findings highlight how multifactorial failures in documentation and system design can compromise patient safety.

Together, the evidence underscores the urgent need for structured, consistent documentation of food and other non‐drug allergies across healthcare settings. Unlike drug allergies, which are supported by well‐established protocols, non‐drug allergens remain poorly defined and inconsistently recorded. In the UK, while clinical incidents involving food and non‐drug allergies are reported, there is no national framework to standardise how they are captured in the incident reporting system, resulting in variation across NHS Trusts and likely leading to underestimation of the number of food and other non‐drug allergy incidents.

To address this gap, we aimed to evaluate how food and non‐drug allergies are currently documented in NHS EPR systems, assess the extent and nature of reported incidents, and identify system‐ and practice‐level challenges that contribute to inconsistent or incomplete documentation.

## 2 Method

### 2.1 Study Design

The aim of this study was to evaluate how food and non‐drug allergies are documented across NHS EPR systems, identify key challenges and inconsistencies and provide evidence to inform the development of national strategies for standardised documentation. Drug allergy data was included to provide a comparator, allowing evaluation of documentation and reporting practices across both established (drug) and underdeveloped (non‐drug) allergen categories. We employed a cross‐sectional, mixed‐methods design using two complementary data sources: (1) a Freedom of Information (FOI) survey sent to all 209 NHS hospital Trusts across the UK, including devolved nations (December 2024) and (2) an online survey distributed to members of the British Society for Allergy & Clinical Immunology (BSACI) (February 2025).

The FOI survey collected institutional‐level data on current allergy documentation practices, EPR availability, training, local guidance and reported clinical incidents related to allergy, including identified contributory factors. The BSACI survey captured user perspectives on systemic usability, coding limitations, challenges in documenting food and non‐drug allergies, suggestions for improvement and views on the need for national guidance.

**FIGURE 1:**
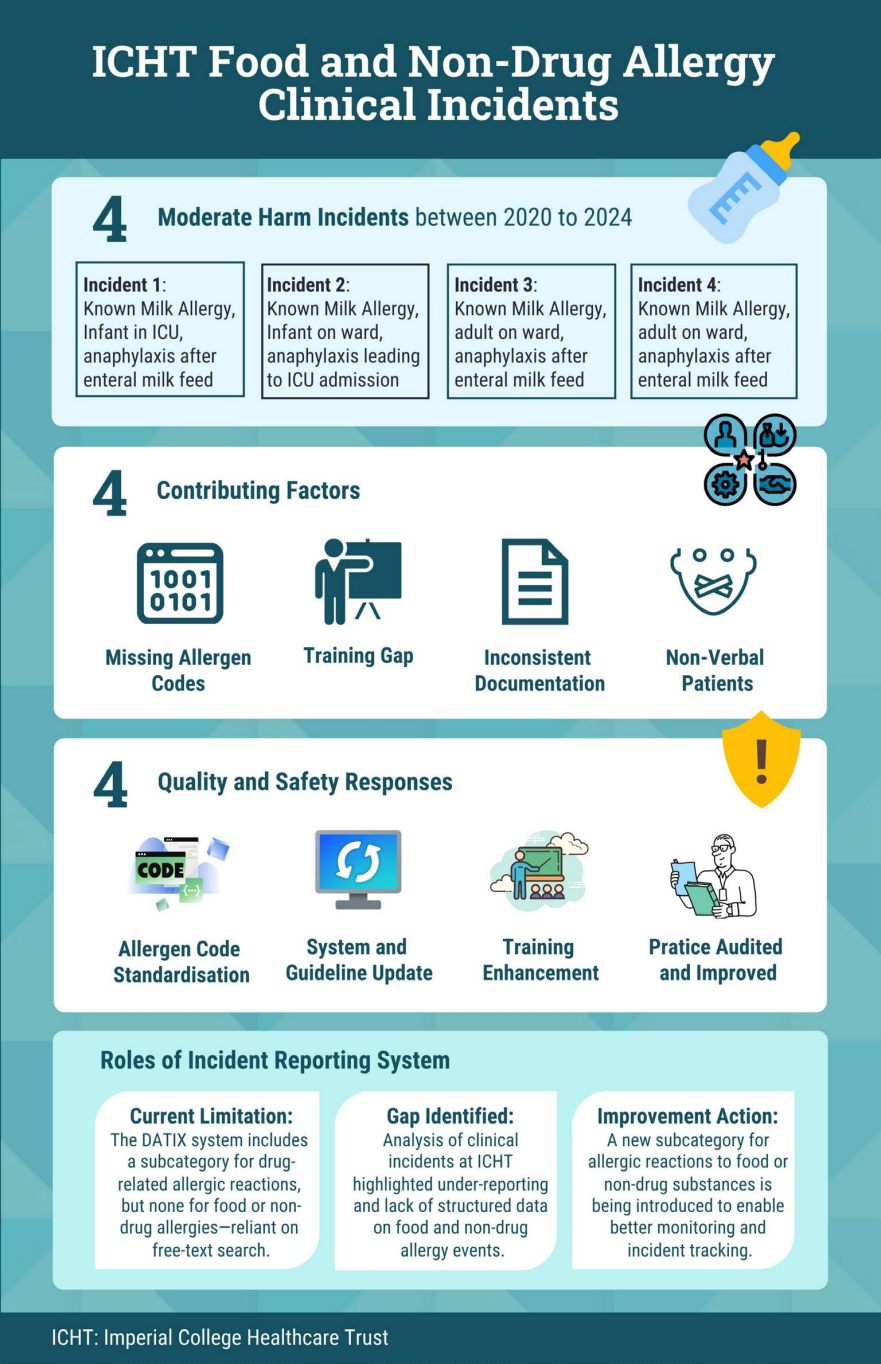
Deep‐dive investigation of ICHT allergy related clinical incidents. Summary of major food and non‐drug allergy‐related clinical incidents at Imperial College Healthcare NHS Trust (ICHT) from 2020 to 2024.

This dual approach provided both system‐wide and frontline insights into current practice. The study design was informed by a detailed local investigation at Imperial College Healthcare NHS Trust, following several moderate‐harm incidents where severe anaphylaxis occurred after known food allergens were administered to allergic patients (Figure 1).

### 2.2 Data Collection

The FOI survey was designed to gather institutional‐level information on EPR systems, documentation protocols, allergy‐related incident reporting and staff training. All 209 NHS hospital trusts were contacted via their designated FOI email addresses, with responses requested within the statutory 20‐working‐day timeframe. A standardised questionnaire containing both quantitative and qualitative items was provided. Initial exploratory work by the local quality and safety team revealed the absence of specific categories for food and non‐drug allergy incidents in existing reporting portals. In response, bespoke search strategies were developed in collaboration with local paediatric and adult allergy teams, as well as the hospital risk management team, and included in the FOI request to support meaningful data retrieval.

Concurrently, the BSACI survey was electronically disseminated to UK‐based members of the society involved in allergy care and EPR documentation. Hosted on Qualtrics, the survey was fully anonymized and included closed‐ and open‐ended questions, accompanied by an information sheet and consent form. Eligible participants were registered healthcare professionals working in the UK, with roles involving allergy management and EPR use. Responses from non‐NHS organisations or individuals not involved in clinical care were excluded. All data were anonymised and securely stored on GDPR‐compliant servers at Imperial College London.

### 2.3 Data Analysis

Quantitative data were analysed using descriptive statistics to assess trends in EPR use, documentation practices and incident reporting. For group comparisons involving non‐parametric data, the Mann–Whitney *U* test was applied. In addition, (median) quantile regression was conducted to explore the relationship between institutional characteristics and incident reporting outcomes, allowing for robust analysis of non‐normally distributed variables. Free‐text responses were analysed thematically to identify common challenges, barriers and suggestions for improving allergy documentation.

### 2.4 Ethics Statement

The FOI component did not require ethical approval, as it involved publicly accessible institutional data. The BSACI survey received ethical approval through the Integrated Research Application System (IRAS) and was reviewed and approved by the Health Research Authority (HRA). All survey participants provided informed consent prior to participation.

## 3 Results

### 3.1 FOI Survey

#### 3.1.1 Response Details

**FIGURE 2:**
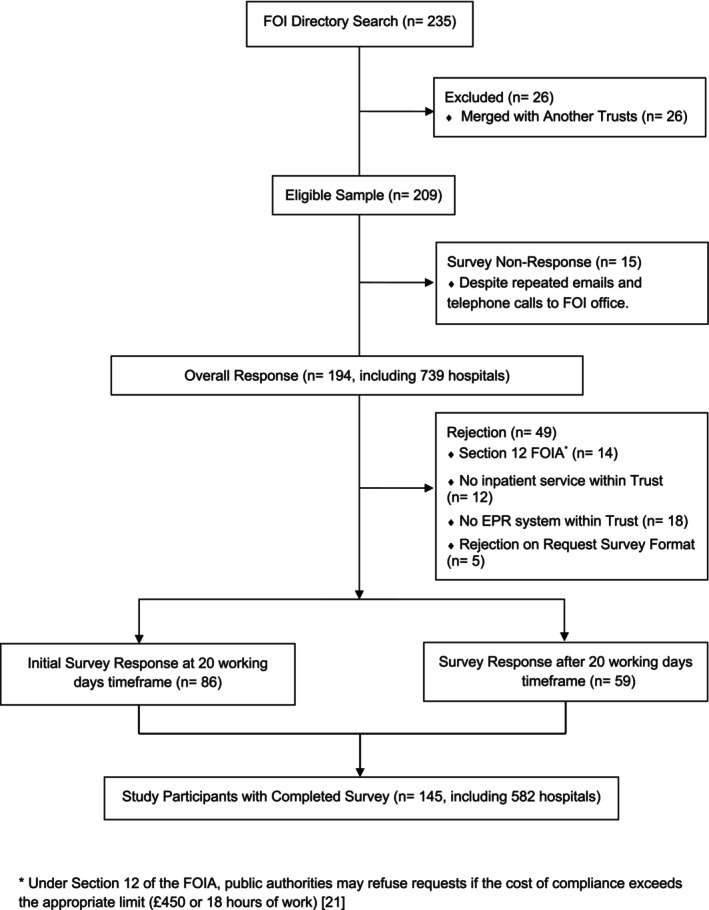
CONSORT flow diagram. Overview of FOI survey participation.

Of 209 eligible NHS Trusts, 86 (41%) replied within the 20‐day statutory limit and 194 (92.8%) eventually responded; 145 (69.4%) completed every question (Figure 1). Forty‐one Trusts invoked FOI Act Section 12 [20, 21] to decline granular incident data but supplied the remainder of the questionnaire (Figure 2).

#### 3.1.2 EPR Penetration and Diversity

**FIGURE 3:**
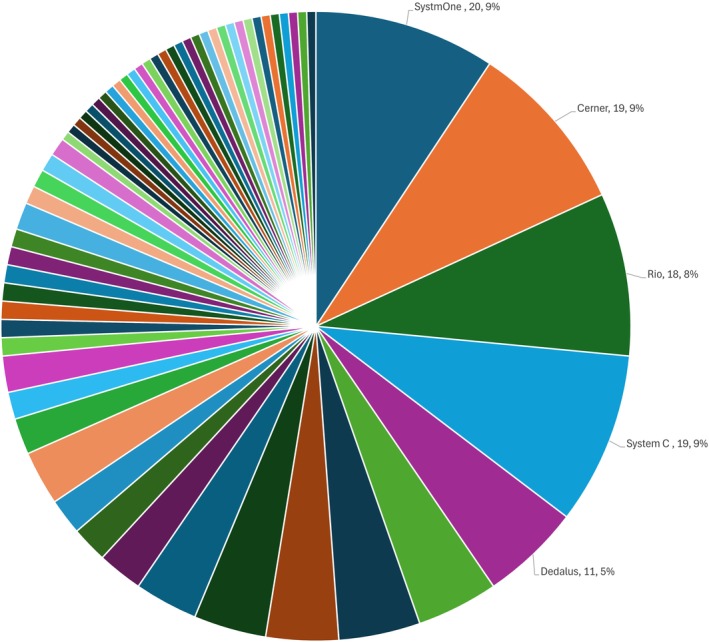
Types of EPR systems used in NHS trusts. A total of 215 responses from 133 NHS Trusts detailed the types of EPR systems in use. The five most frequently reported systems—SystmOne, Cerner, System C, Rio and Dedalus—are highlighted in the pie chart above. Each colour is a separate EPR system (*n* = 61).

Most respondents who completed the survey (133/145, 92%) used an EPR; 12/145 (8%) remained fully paper‐based. Sixty‐one distinct EPR products were named, indicating striking digital heterogeneity (Figure 3 and Table S1). The five commonest platforms were SystmOne, Cerner, System C, Rio and Dedalus, while 44 Trusts ran more than one concurrent system.

##### 3.1.2.1 Allergy Interfaces and Alerts in EPR

Among 120 Trusts answering the interface section, pop‐up warnings (50.8%) were the dominant alert modality, followed by red flags (35%), highlighted text (31.7%), banner displays (12.5%) and others (25%). Notably, 6% reported no electronic allergy flag at all.

##### 3.1.2.2 Training and Local Governance in Allergy Documentation

Mandatory training in allergy documentation was confirmed by 58% (77/133) of Trusts; 27% (36/133) offered optional modules and 15% (20/133) provided none. Of those delivering training, 25.7% (29/113) omitted food or other non‐drug allergens. Local guidelines were present in 60% (80/133) of Trusts, absent in 30% (40/133) and unknown to staff in the remainder. One in four guidelines failed to cover non‐drug allergens.

##### 3.1.2.3 Allergy Clinical Incidents Categorisation and Burden

**FIGURE 4:**
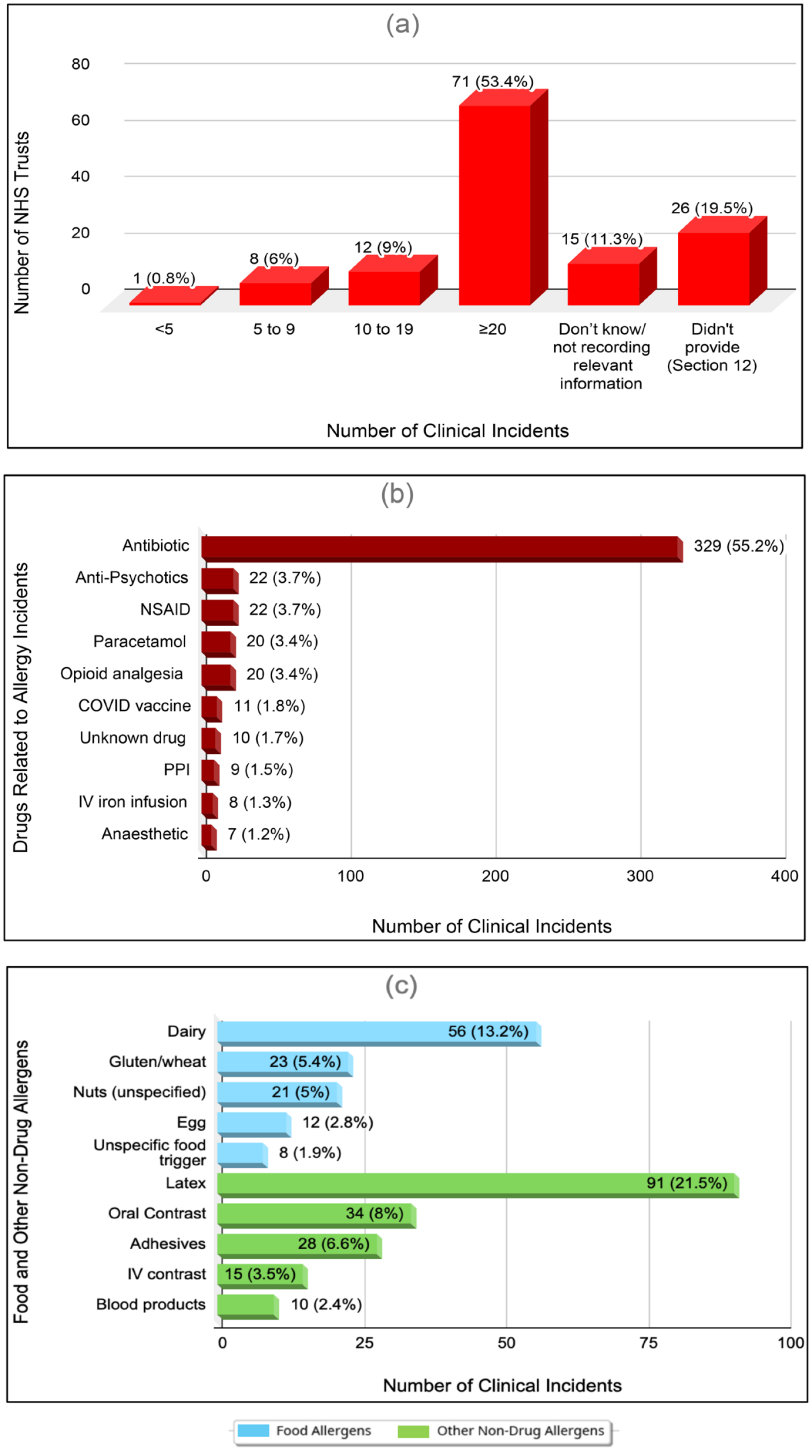
Reported allergy‐related clinical incidents and common allergens. (a) Number of allergy‐related clinical incidents reported by NHS Trusts over the past 10 years. (b) Top 10 drug‐related allergens associated with clinical incidents. (c) Top 10 food and non‐drug allergens related to reported incidents.

More than half of Trusts (72/133, 54%) had no dedicated category in their incident‐reporting system for food or other non‐drug allergy events. Over the decade 2014–24, 112 Trusts (84%) recorded at least one allergy‐related incident; 53% logged ≥ 20 (Figure 4a). Quantitative returns from 92 Trusts detailed 12,385 incidents up to the last 10 years: drugs 7724 (62%), food and other non‐drug allergens 1277 (11%) and 3384 unclassified (27%).

##### 3.1.2.4 Drug‐Allergy Incidents

Seventy Trusts supplied narrative summaries (up to 10 incidents each) based on severity, prioritised in chronological order, yielding 596 cases (Table 1). Adults accounted for 89%; most events caused low harm (56%). Antibiotics were involved in 55% of cases, led by penicillins (43% of antibiotic incidents), followed by NSAIDs and antipsychotics (Figure 4b). Four deaths were reported, attributed to chlorphenamine, clopidogrel, clozapine and nitrofurantoin.

**TABLE 1:** Demographic information of clinical incidents related to allergy.

	Drug allergies related clinical incidents (*n* = 596)	Food and other non‐drug allergies related clinical incidents (*n* = 424)
Age distribution		
Adult (≥ 17 years old)	532 (89.3%)	352 (83%)
Paediatric (< 17 years old)	34 (5.7%)	53 (12.5%)
Unspecified	30 (5%)	19 (4.48%)
Level of harm		
No harm	142 (23.8%)	144 (33.9%)
Low harm	334 (56%)	222 (52.3%)
Moderate harm	101 (16.9%)	48 (11.3%)
Severe harm	15 (2.5%)	9 (2.1%)
Death	4 (0.7%)	1 (0.2%)

*Note:* This table presents the age distribution and associated levels of harm for clinical incidents related to drug allergies (*n* = 596) and food or other non‐drug allergies (*n* = 424). Incidents are categorised by patient age group (adult ≥ 17 years, paediatric < 17 years and unspecified) and severity of harm (no harm, low harm, moderate harm, severe harm and death). Percentages represent the proportion within each category.

##### 3.1.2.5 Food and Other Non‐Drug Allergy Incidents

Sixty‐three Trusts provided 424 detailed cases (Table 1). Low‐harm outcomes predominated (52%), with adults comprising 83% and paediatric patients 12.5%, proportionally higher than in drug events. Dairy was the leading food culprit (13.2%), while latex was the leading non‐drug trigger (21.5%) (Figure 4c). Nine cases caused severe harm—five from oral contrast agents, two from latex and one each from dairy and cashew; the single fatality followed contrast exposure. Across all 280 analysable narratives, the commonest reactions were anaphylaxis (11%), local skin signs (7%) and oral tingling (5%). Reaction type was undocumented in 54% of records. Thirty‐one anaphylaxis cases were identified; 48% of the cases were coded as ‘no’ or ‘low’ harm despite meeting national ‘moderate‐harm’ criteria.

Forty‐four Trusts identified potential causes. Top issues (each 20.5%) were: allergy entered but not flagged; failure to consult the allergy section; and wider system failures. Mis‐labelling, outdated entries, alert fatigue, kitchen cross‐contamination and missed radiology checks also emerged.

##### 3.1.2.6 Subgroup Analysis

**FIGURE 5:**
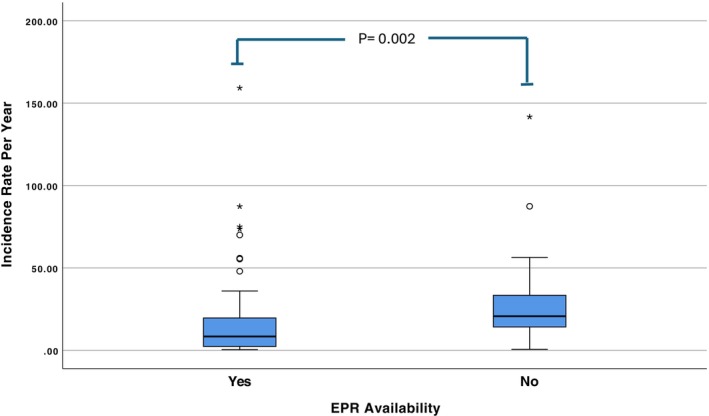
Boxplot comparing the annual clinical incident rates between Trusts with and without EPR systems, based on Mann–Whitney *U* test. The Non‐EPR Trusts have a higher rate of incidents per year (median: 20.7 compared to 8.4) and are statistically significant (*p* = 0.002). Follow‐up requests to initially non‐responding non‐EPR Trusts yielded additional responses, bringing the total to *n* = 19. The horizontal line in the box indicates the median. Dots and asterisks represent the outliers.

1. Trusts Using EPR and Those Without: Initially, 92 out of 145 Trusts provided incident counts. A follow‐up request to previously rejected non‐EPR Trusts yielded 11 additional responses, though these provided only incident counts. This resulted in final totals of 84 EPR Trusts and 19 non‐EPR Trusts with incident data. Trusts without an EPR system reported significantly more allergy‐related clinical incidents (median: 204; *n* = 19) than those with EPR (median: 69.5; *n* = 84). The median annual incident rate was 20.7 (range: 0.6–141.7) for non‐EPR Trusts vs. 8.4 (range: 0.4–159.2) for EPR Trusts (*p* = 0.002; Figure 5) representing a 143% higher rate without EPR.

##### 3.1.2.7 Median Quantile Regression

The median quantile regression analysis (*τ* = 0.5) revealed that EPR availability was significantly associated with the annual rate of allergy‐related clinical incidents (Table 2). Specifically, NHS Trusts without EPR systems reported approximately 11.6 more incidents per year compared to those without, controlling for population catchment size and deprivation level (*β* = 11.573, *p* = 0.015). In contrast, population catchment size showed a negative but non‐significant relationship with incident rates (*β* = −2.069E–6, *p* = 0.432), suggesting that larger populations might be associated with slightly fewer reported incidents, although the evidence was not statistically conclusive. Similarly, deprivation level (IMDD) was not significantly associated with incident rates (*β* = 0.572, *p* = 0.452).

2 Single versus multiple EPRs: Institutions running multiple platforms (*n* = 35) logged 31% more incidents per year than those with a single system (*n* = 47, median 10.7 vs. 8.2, *p* = 0.82).
3 Local guideline status: Median annual incident rates were 10.6 (guideline present, *n* = 51), 7.9 (none, *n* = 23) and 15.5 (staff unsure, *n* = 8).
4 In‐house allergy service: Of the 82 Trusts that provided data on clinical incident counts, 50% reported having an in‐house paediatric or adult allergy service. Trusts hosting a paediatric or adult allergy service (41/82) recorded twice as many incidents per year as those referring externally (22.3 vs. 11.0; *p* = 0.003; see Figure S1).

**TABLE 2:** Median quantile regression results predicting the annual incidence rate of allergy‐related clinical events.

Parameter	Coefficient	SE	Significance (*p*)	95% confidence interval (lower and upper bound)	
(Intercept)	−3.442	8.0243	0.915	−19.376	12.493
EPR availability	11.573	4.6631	0.015	2.313	20.833
Population catchment by trust	−2.069E‐6	2.6220E‐6	0.432	−7.276E‐6	3.138E‐6
IMDD	0.572	0.7573	0.452	−0.932	2.076

*Note:* The model includes EPR availability (presence of an electronic patient record system in the NHS Trust), population catchment (total population served by the Trust) and IMDD (Index of Multiple Deprivation Decile) as predictors.

##### 3.1.2.8 Thematic Analysis on Challenges and Suggestions From FOI Respondents

Five recurrent challenges were identified, including system‐level issues, poor usability, limited interoperability, data quality concerns and alert system shortcomings. Suggestions for improvement focused on standardisation, training, enhanced system design and better data sharing. Detailed themes and descriptions are outlined in Table 3.

**TABLE 3:** Challenges and suggested improvements in allergy documentation and management.

Theme	Description
Challenges	
1. Systemic and organisational issues	Inconsistent documentation practices, unclear policies for updating allergy records, insufficient staff training and awareness
2. Usability and interface concerns	Cumbersome data entry, limited fields for detailed information, difficulty distinguishing allergies from other adverse reactions
3. Interoperability and data sharing	Data loss during transitions, inconsistent coding standards and lack of full system integration
4. Data quality and standardisation	Use of free‐text entries, incomplete or outdated records, limited terminology and coding options
5. Clinical decision support and alerts	Alert fatigue, missed/inappropriate alerts, poor integration into clinical workflows
Suggestions for improvement	
1. Standardisation	Mandate standardised coding, develop national guidance, consistently record allergen details including severity and reaction type
2. Training and education	Nationwide mandatory training, education on distinguishing allergies from intolerances/sensitivities, targeted resources for accuracy
3. System usability	Redesign EPRs for structured data entry, implement intelligent alerts, add mandatory allergy prompts, improve navigation
4. Interoperability	Establish centralised allergy database, enable cross‐sector data sharing, ensure real‐time synchronisation across care settings

*Note:* A thematic summary of challenges and recommended improvements in allergy documentation and management is listed.

### 3.2 BSACI Survey

#### 3.2.1 Response Details

Between February and April 2025, 117 of 1127 members responded (10.4%); 59% completed every question. Most were London‐based (44%), with representation from the majority of English regions and the devolved nations (Figure S2).

#### 3.2.2 Respondent Experiences With Allergy Documentation in EPR Systems

Of 103 clinicians, 55% said they ‘always’ or ‘most of the time’ enter non‐drug allergies in the EPR, while 45% stated they ‘sometimes’ or ‘rarely/never’ recorded a non‐drug allergy. Food allergies were recorded by 80%, latex 46%, environmental allergens 26% and insect stings 22%. For food reactions, 46% documented all IgE‐mediated allergies and 41% both IgE‐ and non‐IgE‐mediated forms; 12% never entered food allergies.

On usability, 37% found allergy documentation difficult or very difficult; only 30% found it easy. 39% reported problems locating suitable codes for non‐drug allergens.

Respondents highlighted multiple challenges in documenting non‐drug allergies in EPR systems, which were grouped into six main themes (Table S2). These included usability and search limitations of allergen codes, incomplete and poorly structured allergen lists, coding gaps and reliance on free text, inadequate support for reaction type documentation, weak allergy alert integration and environmental and other non‐food allergen challenges.

#### 3.2.3 Documentation and Coding Accessibility for Food and Other Non‐Drug Allergens in EPR Platforms

Fewer than half (43/96, 45%) EPR platforms could readily identify the 14 UK‐mandated food allergens in their EPR. Where lists were incomplete, 80% of respondents resorted to free text. Only 55% of systems allowed simultaneous entry of a food allergy and a ‘no known drug allergy’ (NKDA) status, owing to conflicts in rule logic or screen design.

When documenting multiple allergies, two‐thirds recorded each allergy separately; one quarter summarised multiple allergens in a single line or free‐text summary.

#### 3.2.4 Suggested Enhancement by BSACI Members

Priority requests were enhancing allergen databases (67%), streamlined user interfaces (64%), integration of allergy data with clinical decision‐support (49%) and broader coding options (48%). Additional themes concerned workflow efficiency, structured allergy reaction record fields, improved alert logic and staff education. The majority of 90% supported the development of national standards for documenting food and other non‐drug allergies.

## 4 Discussion

This study presents the first national analysis of food and non‐drug allergy documentation across NHS Trusts, combining FOI data with frontline input from BSACI members. Findings reveal significant variation in documentation practices, EPR functionality and clinical governance, highlighting the need for national standardisation.

While 90% of Trusts reported having allergy sections in their EPR systems, a detailed review showed inconsistent methods for recording and flagging food and non‐drug allergies. With over 60 EPR platforms in use, many within the same Trust, this fragmentation undermines data consistency, interoperability and patient safety, reinforcing concerns noted in existing literature [10, 11].

The findings from the median quantile regression analysis indicate a significant association between the availability of EPR and the annual rate of allergy‐related clinical incidents. Specifically, NHS Trusts without EPR systems experienced a higher number of incidents, approximately 11.6 more incidents per year, than those with EPR systems, even after accounting for population size and deprivation level. This suggests that EPR implementation may play a meaningful role in reducing such incidents. Importantly, having an EPR alone was insufficient. Users felt that improved functionality such as integrated allergy alerts and better staff training could further improve safety and documentation.

We identified that incident reporting systems frequently do not feature a specific category for food and non‐drug allergy‐related incidents. This necessitated free‐text searches, and relevant incidents were probably missed, resulting in an underestimation of the frequency of food/non‐drug‐related events.

Unexpectedly, Trusts offering training reported a higher incident rate. This potentially reflects improved recognition and reporting rather than a true increase in events, indicating that effective training may enhance detection and play a preventive role. This finding aligns with Louis et al., who observed a rise in reported patient safety events following a targeted educational intervention among residents and teaching faculty [22]. Training fostered a culture of safety, reduced misunderstandings and promoted open communication, ultimately making staff more comfortable reporting errors [23].

Anaphylaxis was reported but often misclassified as ‘no/low harm,’ contradicting national safety definitions [24]. This reflects inconsistent allergy training and recognition of anaphylaxis and its severity across the UK, highlighting a clear need for targeted education.

Despite these, only 58% of Trusts mandate allergy training, and just 60% have local guidelines, revealing a governance gap. Moreover, while 60% of Trusts reported having allergy documentation protocols, only 35% of BSACI allergy practitioner respondents were aware of them, suggesting a potential disconnect between policy and practice.

Trusts with in‐house allergy services reported significantly higher incident rates, likely due to better recognition and reporting supported by specialist teams and clear pathways. In contrast, Trusts without such services may underreport due to limited expertise. These findings highlight the benefits of integrated allergy services to improve care quality and safety monitoring.

Thematic analysis of BSACI responses highlighted recurring documentation challenges, including incomplete allergen databases, poor search tools, inconsistent terminology and reliance on free‐text. These issues weaken clinical decision support, contributing to underreporting and delayed recognition. Poor functionalities, such as cluttered drop‐down menus and the inability to record food allergies alongside NKDA, further compromise safety.

These findings support prior research highlighting the need for accurate, structured allergy documentation. Li et al. showed that cleaning hospital allergy records removed thousands of errors [11], while US data link most allergy safety events to documentation flaws, human error and weak alert systems [13]. There is limited literature exploring the relationship between documentation of food and non‐drug allergies and the occurrence of clinical incidents within hospital settings. Tejedor‐Alonso et al. reported that approximately 14.7% of anaphylaxis cases in hospitalised patients were attributed to food, non‐drug triggers or were idiopathic [9], while several other studies have primarily focused on non‐drug allergy incidents within perioperative environments [14]. However, these investigations often remain narrowly focused. In contrast, our study provides a broader and more comprehensive view, highlighting the range of potential food and non‐drug allergy‐related clinical incidents that can occur throughout the hospital system.

The qualitative data also revealed strong support for national guidance. An overwhelming 90% of BSACI respondents favoured the development of standardised documentation protocols for food and non‐drug allergies. Suggested solutions included a centralised allergy database, structured coding aligned with Systematised Nomenclature of Medicine Clinical Terms standards and real‐time data sharing across healthcare settings. These align with international calls for harmonisation of allergy documentation as a strategy to reduce preventable harm and improve continuity of care [11, 12].

A multi‐pronged strategy is required to address the systemic deficiencies identified in allergy documentation across NHS EPR systems. Firstly, national standardisation should be prioritised through the development of unified guidelines and structured coding frameworks for food and non‐drug allergens, aligned with SNOMED CT [25] and NICE principles [6]. Secondly, collaboration with EPR suppliers is essential to enhance interoperability, ensure comprehensive allergen lists and optimise alert functionality to minimise reliance on free text and reduce alert fatigue. Thirdly, workforce capability must be strengthened through mandatory, standardised training on allergy documentation and anaphylaxis recognition, integrated into staff induction and continuing professional development. Finally, the introduction of standardised incident reporting categories for food and non‐drug allergies would facilitate national surveillance, benchmarking and shared learning. Together, these measures could improve consistency, data integrity and patient safety across NHS Trusts. Aligning these actions with the NHS digital transformation plan [26] would help embed allergy safety within broader system reform, ensuring that the prevention of avoidable harm from food and non‐drug allergens becomes a core component of the national digital health strategy.

On the other hand, future EPR allergy systems should evolve beyond binary labels to incorporate verification status, supporting evidence and risk level. Structured fields distinguishing *suspected*, *verified* and *de‐labelled* allergies, linked to test results or clinic documentation, could enhance both accuracy and patient safety. Integrated risk‐stratification tools could support penicillin allergy testing and de‐labelling, while maintaining hard‐stop alerts for confirmed severe reactions. Embedding antimicrobial stewardship logic aligned with the WHO AWaRe framework [27] would encourage safe first‐line antibiotic use and reduce unnecessary broad‐spectrum prescribing. Regular review prompts, interoperability across care settings and patient‐accessible updates would ensure records remain accurate and current. This evidence‐based, dynamic approach would balance patient protection from genuine allergy risks with the need to avoid inappropriate antibiotic restriction.

### 4.1 Strengths and Limitations

A key strength of this study is its dual methodology, combining data from nearly 70% of NHS Trusts with insights from frontline clinicians. This provides a robust view of both policy and practice‐level barriers to allergy safety. Thematic analysis further enriches the findings by highlighting user‐reported system limitations.

However, several limitations exist. FOI data may be incomplete or subject to interpretation bias, with 28% of Trusts withholding incident details under Section 12 exemptions. Overall, we achieved strong representation across all UK devolved nations, with participation from 124 Trusts in England, 11 in Scotland, 5 in Wales and 5 in Northern Ireland. The BSACI survey, while valuable, was voluntary and potentially subject to selection bias, particularly as it invited allergy‐interested respondents. Geographic clustering in London also limits generalisability, and duplicate reporting of incidents from the same Trust cannot be ruled out (see map attached in Figure S2). Additionally, evolving clinical practices may reflect temporary rather than systemic issues, and the lack of independently verified incident details limits conclusions about patient outcomes and safety impact.

### 4.2 Clinical Implication

This study highlights the urgent need for national guidance on allergy documentation that extends beyond drug allergies to encompass food and other non‐drug allergens. Key policy implications include the integration of mandatory training, structured EPR templates, standardised coding libraries and improved alert functionality. System vendors and NHS Digital stakeholders must address interoperability and design limitations that undermine documentation accuracy and patient safety—aligning these improvements with the NHS's 10‐year digital transformation plan [26].

## 5 Conclusion

Inconsistent and unstructured documentation of food and non‐drug allergies in NHS EPR systems presents a significant patient safety risk. This study provides compelling evidence for the development of national guidelines, standardised training and system‐level reforms to ensure safer, more consistent allergy documentation. By aligning documentation and training practices for food and non‐drug allergens with those already established for drug allergies, healthcare systems can better protect patients from avoidable harm and improve the reliability of electronic records in clinical care.

## Author Contributions

M.L.Q. conceptualised the study, acquired the data, performed data analyses, contributed in result interpretation and drafted the initial manuscript; E.H. conceptualised the study, assisted in result interpretation, critically reviewed and revised the manuscript; C.G. conceptualised the study, assisted in result interpretation, critically reviewed and revised the manuscript; and all authors approved the final manuscript as submitted and agree to be accountable for all aspects of the work.

## Funding

The authors have nothing to report.

## Conflicts of Interest

M.L.Q. and E.H. declare no conflicts of interest. C.G. declares that she is the honorary secretary at BSACI (Trustee).

## Supporting information


**Data S1:** Supporting Information S1

## Data Availability

The data that support the findings of this study are available from the corresponding author upon reasonable request.
