# Supplementary material for: Documentation of Drug and Non‐Drug Allergies in Public Hospital Electronic Health Records: A National Survey
Source: Clin Exp Allergy. 2025 Dec 2;56(1):41–52. doi: 10.1111/cea.70186 (PMC12774576; doi:10.1111/cea.70186)
Supplement: Supplementary file 1 — Data S1: Supporting Information S1 [file CEA-56-41-s001.docx]

**Supplementary Material**

**Title: Documentation of Drug and Non-Drug Allergies in Public Hospital Electronic Health Records: A National Survey**

Author: Dr. Melvin Lee Qiyu^1,2^, Dr. Claudia Gore^1,3^ and Dr. Erika Harnik^3^

1. Section of Inflammation, Repair and Development, National Heart and Lung Institute, Imperial College London, London, UK
2. Dept. Paediatric Allergy and Respiratory Medicine, University Hospital Southampton NHS Foundation Trust, Tremona Rd, Southampton SO16 6YD.
3. Dept. Paediatric Allergy, Imperial College Healthcare NHS Trust, St. Mary’s Hospital, Praed Street, London W2 1NY.

| No | Content | page |
| --- | --- | --- |
| Suplementary methods | A: Codebook: Challenges in Allergy Documentation in EPR Systems | [2](#Page2) |
|  | B: Codebook: Improving Allergy Documentation in EPR Systems | [3](#Page3) |
| TABLE S1 | Detailed Portions of Each EPR System Used in NHS Trusts | [4](#Page5a) |
| table s2 | Thematic Analysis on Challenges Encountered by Respondents Regarding Documentation of Non-Drug Allergies in EPR | [5](#Page5c) |
| Figure s1 | Boxplot Comparing Annual Clinical Incident Rates between Trusts With and Without In-House Allergy Services, Based on Mann-Whitney U Test | [6](#Page6a) |
| Figure S2 | BSACI Member Survey Respondents Geographic Distribution | [6](#Page6b) |
| other s1 | Integrated Research Application System (IRAS) Approval Letter | [7-9](#Page7) |
| Other s2 | FOI Survey | [10-15](#page9) |
| Other s3 | BSACI Member Survey | [16-22](#Page15) |

**Supplementary Methods:** Supplementary Methods A and B provide the tables of codebook that was conducted during the process of generating thematic analysis core themes.

**A. Codebook: Improving Allergy Documentation in EPR Systems.** *This codebook outlines the analytical process used to derive codes and themes from free-text responses, focusing on suggested improvements to allergy documentation practices across NHS Trusts.*

**B. Codebook: Challenges in Allergy Documentation in EPR Systems.** *This*

*codebook outlines the analytical process used to generate codes and themes from free-text*

*responses, capturing the key challenges reported by NHS Trusts during thematic analysis.*

**Table S1. Detailed Portions of Each EPR System Used in NHS Trusts.** *This table outline the detailed counts of individual EPR system usage by the respondents.*

**Table S2. Thematic Analysis on Challenges Encountered by Respondents Regarding Documentation of Non-Drug Allergies in EPR.** *Six key themes were identified through analysis, each outlining specific challenges encountered in documenting 14 common food and other non-drug allergies, as detailed in the table.*


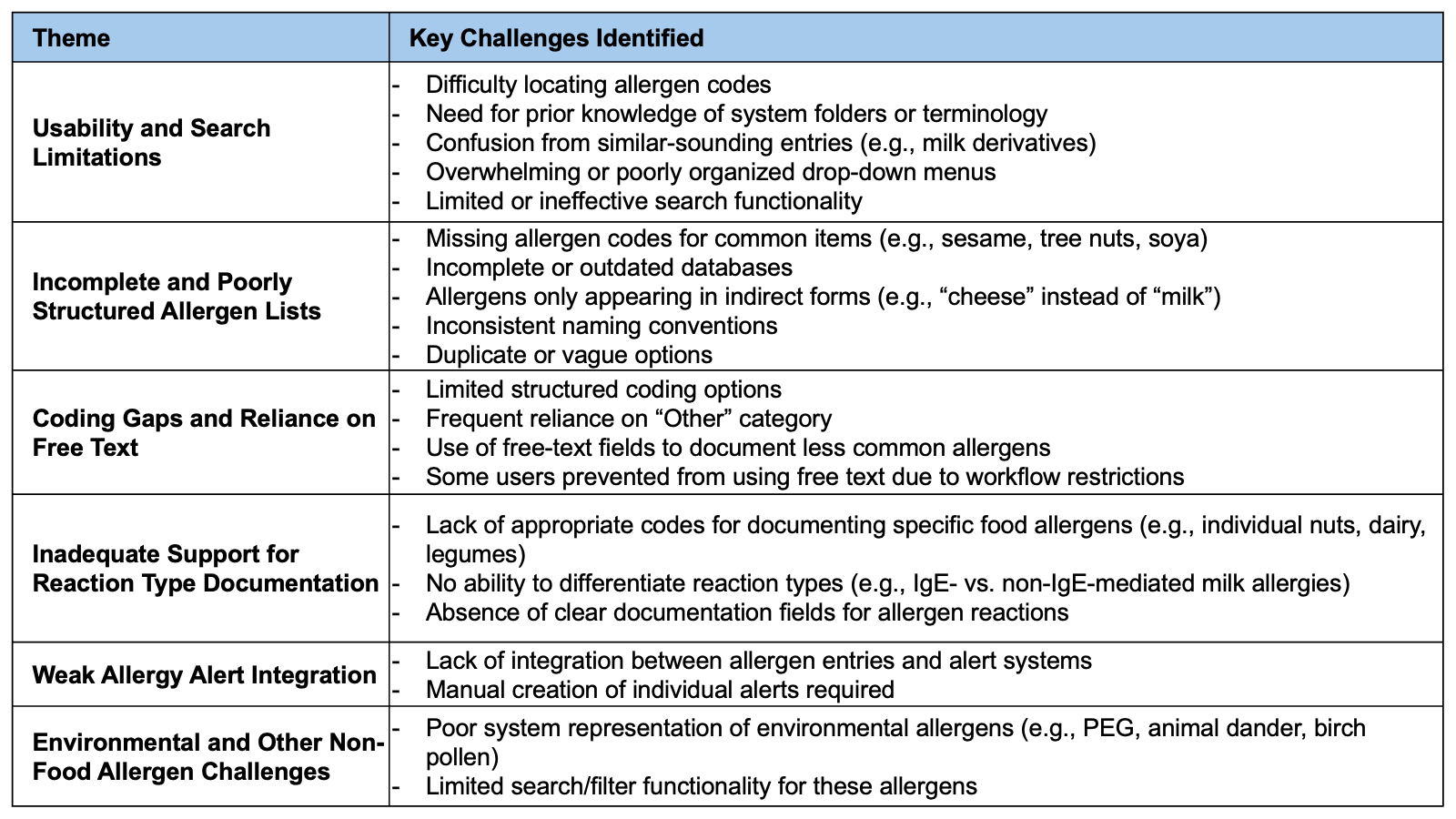


**Figure S1. Boxplot co****mparing annual clinical incident rates between Trusts with and without in-house allergy services.** *Trusts with in-house services reported significantly higher incident rates (median: 22.3) than those without (median: 11.0), based on Mann-Whitney U test (p = 0.003). The horizontal line within each box indicates the median; dots and asterisks represent outliers.*

**Figure S2. BSACI Member Surve****y Respondents Geogra****phic Distribution**


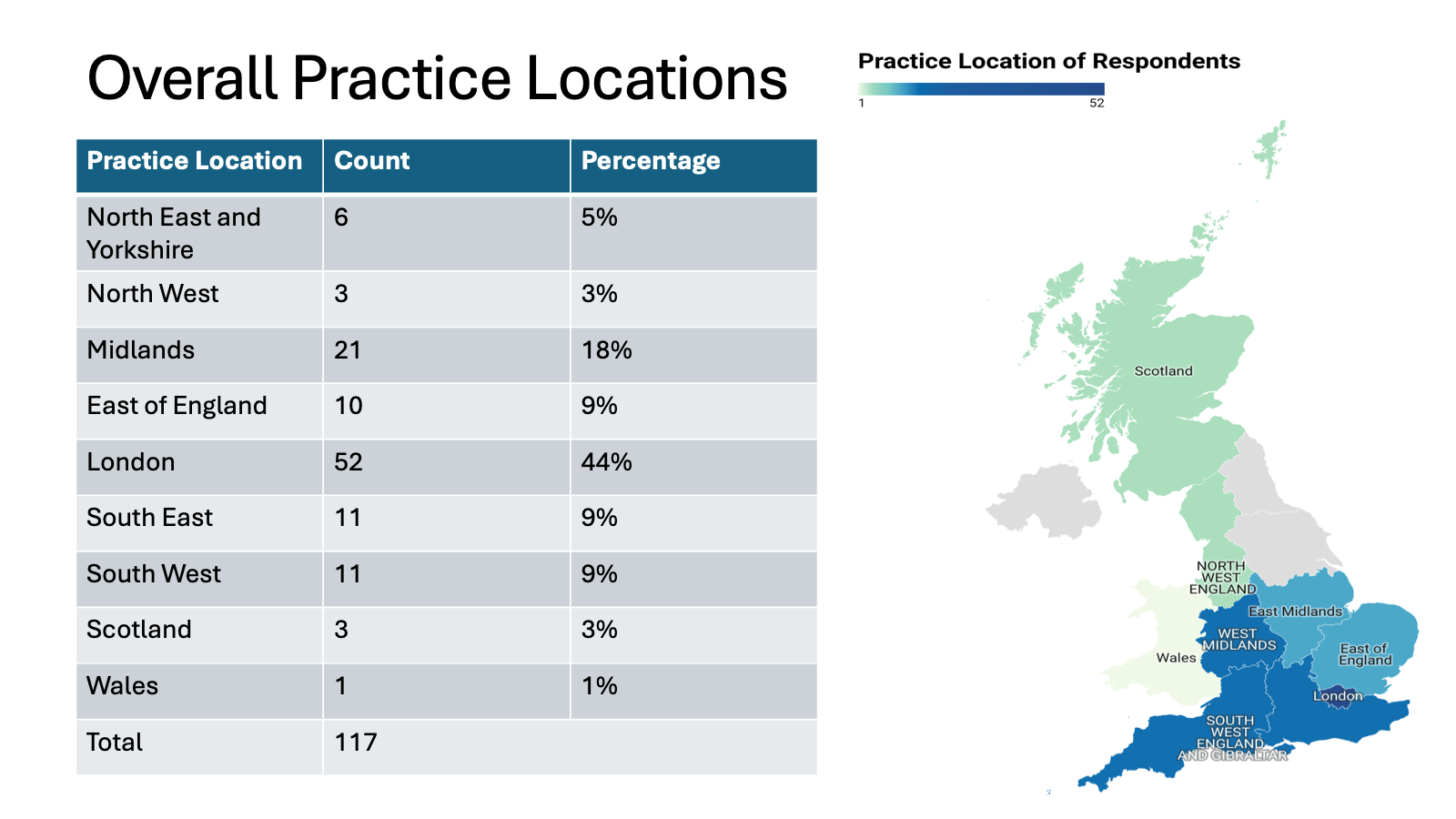


**Other S1. Integrated Research Application System (IRAS) Approval Letter**

**Other S2. Freedom of Information (FOI) Survey**

Freedom of Information Request on Allergy Recording in Electronic Patient Records

Survey Flow

Block: Section 1: General Information (7 Questions)

Standard: Section 2: Allergy Recording System (10 Questions)

Standard: Section 3: Allergy incidents (11 Questions)

Standard: Section 4: Feedback and Improvements (2 Questions)

Cover Statement  Freedom of Information (FOI) Request on Allergy Recording in Electronic Patient Records (EPR) in NHS Trusts

INTRODUCTION

Who are we?
Imperial College London are undertaking a study looking at documentation of food, drug and non-drug allergies within electronic patient records and any related allergy patient safety incidents in hospital. 

Why are we asking for this information?
We suspect that that awareness and documentation of food and non-drug allergies in Electronic Patient Records (EPR) are insufficient and pose a risk to patient safety. We are gathering information on how food, drug, and non-drug allergies are documented and managed within electronic patient records (EPR) across different NHS Trusts in the UK. This data will help identify current practices, challenges, and areas where national guidelines could be improved or introduced to improve patient safety and care.

Your information is fundamental in helping us understand the variations in allergy documentation practices across NHS Trusts. By sharing your experiences and insights, you will contribute to potential improvements in allergy management and patient safety on a national level.

Who can take part?
The survey link was distributed through the FOI email address to all NHS Trust Foundations and Health Boards. The selection of participants will be determined by the FOI inbox manager, with the ideal participant being someone who has access to the incident reporting portal.

How long will it take to complete this survey?
The survey should take approximately 10-15 minutes to complete. It may take longer to gather the information needed for the survey via your incident reporting system. We appreciate your time and effort in providing valuable information.

Thank you for your participation and valuable contribution to improving allergy management and patient safety.

Q1 Trust Name:

________________________________________________________________

Q2 Type of Healthcare Facilities

- District General Hospital (DGH) (1)
- Specialty Hospital (2)
- Private Hospital (3)
- Community Hospital (4)
- Other (Please Specify) (5) __________________________________________________

Q3 Demographic of Hospital Care

- Adult Hospital (1)
- Paediatric Hospital (2)
- General Hospital (Both paediatric and adult) (3)
- GP surgery (4)
- Other (Please Specify) (5) __________________________________________________

Q4 Respondent’s Role in the Trust:

- Medical Records Manager (1)
- IT Specialist (2)
- Clinician (3)
- Administrator (4)
- Other (Please Specify) (5) __________________________________________________

Q5 Does your Trust use electronic patient records (EPR)?

- Yes (1)
- No (2)

Q6 Which EPR system does your Trust use?

- Cerner (1)
- Epic (2)
- System C (3)
- Dedalus (4)
- Altera (5)
- Other (Please Specify (6) __________________________________________________

Q7 Does the EPR system used by your Trust include a specific section for recording food, drug, latex, and other allergies?

- Yes (1)
- No (2)

Q8 If yes to question 7, how is the initial allergy information typically entered into the system? (Select all that relevant)

- Manually by Doctor (1)
- Manually by Pharmacist (5)
- Manually by Nurse (6)
- Manually by Dietitian (7)
- Automatically from Previous Records (2)
- Manually by Administrative Staff (3)
- Other (Please Specify) (4) __________________________________________________

Q9 If yes to question 7, who is responsible for updating and/or checking allergy information in the patient's electronic record? (Select all that apply)

- Clinicians (e.g., doctors, nurses) (1)
- Administrative Staff (2)
- Pharmacists (3)
- IT/Technical Support Staff (4)
- Don’t Know (5)
- Other (Please Specify) (6) __________________________________________________

Q10 How is the allergy information flagged or highlighted in the patient’s records to alert healthcare providers?

- Red Flag (1)
- Pop-up Alert (2)
- Highlighted Text (3)
- Other (Please Specify) (4) __________________________________________________
- Not highlighted/ alerted on the system (5)

Q11 What training, if any, is provided to staff on the correct recording of allergies in patient records?

- Mandatory Training Sessions (1)
- Optional Training (2)
- No Training Provided (3)
- Other (Please Specify) (4) __________________________________________________

Q12 If training is provided on allergy documentation, does it specifically cover different types of allergies in the training materials?

- Only drug allergy recording (1)
- Both drug and non-drug allergy recording (2)
- Drug, food, and other non-drug allergy recording (e.g., latex) (3)
- Don't know/ Unsure (4)

Q13 Does your Trust have a Local Guideline or Standard Operating Procedure (SOP) in place covering allergy documentation on the EPR?

- Yes (3)
- No (5)
- Don't know/ Unsure (4)

Q14 If yes to Question 13, does this guideline/SOP include documentation for allergens below? (Select all that relevant)

- Drugs (1)
- Food (2)
- Other non-drug substances (e.g. latex) (3)
- Don't know/ Unsure (5)

Q15  Does your hospital have access to specialist allergy advice for paediatric patients?

- Yes, please specify if this service available is available through In-House, Local Centre or Regional Centre. (1) __________________________________________________
- No (2)

Q16 Does your hospital have access to specialist allergy advice for adult patients?

- Yes, please specify if this service available is available through In-House, Local Centre or Regional Centre. (1) __________________________________________________
- No (2)

Section 3: Incident Section 3: Patient Safety Incidents In this section, we would like to gather some information about patient safety incidents related to allergies in hospital, for example patients who have been administered penicillin antibiotics when they have a penicillin allergy. We would like information on up to 10 cases each for both drug allergy and food or non-drug allergy incidents, prioritised by severity of harm, followed by the most recent incidents.

Our local risk team recommends that you gather the following information for your incident reporting system before answering the following questions:

1. Drug allergy incidents- Allergen, Age, Level of harm
2. Food and other non-drug allergy incidents- Allergen, Age, Reactions, If reported as serious incident, Level of harm, Is the allergen previously documented in patients' note, Is the the allergen correctly documented on EPR
3. Common causes identified on food and other non-drug allergy incidents reported. 

Tips:
We recognize that many Trusts may not have a specific category for food and other non-drug allergies in their incident reporting portals. However, we have identified a few related categories that are often associated with the documentation of these incidents, including:
1. Food allergens incidents:
- Insufficient help with eating and drinking
- All other medication incidents (errors with prescribing, administration, follow-up etc.)

2. Medication allergen incidents:
- All other medication incidents (errors with prescribing, administration, follow-up etc.)
- Other injury/accident
- Inadequate or inappropriate medical care    

3. Other search terms including- "anaphylaxis", "allergy", "food allergy", "allergic", "urticaria", "urticarial", "hives", "angioedema", "anaphylactic", "non-drug allergy", "adrenaline", "wheezing", "stridor", "EpiPen", "antihistamine"

4. Consider other search terms for non-drug allergy incidents including "Latex" , "Chlorhexidine" , "Povidone iodine" , "Macrogol", "PEG-polyethylene glycol" , "Polysorbate 20", "Polysorbate 80" , "Mannitol" , "EDTA" , "Tromatemol", "Trismatemol", "Metacresol" , "Arginine"

Q17 Does the incident reporting platform have a specific category for recording food or other non-drug allergy incidents?

- Yes (1)
- No (2)

Q18 In the last 10 years, has your Trust recorded any incidents where a patient was administered a food, drug, or other substance (e.g., latex) they were known to be allergic to?

- Yes (1)
- No (2)

Q19 If yes to question 18, how many such incidents have been reported in the last 10 years? [Numerical Response]

- <5, please specify (1) __________________________________________________
- 5 - 9, please specify (2) __________________________________________________
- 10 - 19, please specify (3) __________________________________________________
- ≥ 20, please specify (4) __________________________________________________
- Don't know (5)

Q20 If yes to question 18, please indicate the number of incidents for each category: [Numerical Response]

- Drug allergy incidents (1) __________________________________________________
- Food allergy incidents (2) __________________________________________________
- Incidents to other allergic substances (3) __________________________________________________
- Don't know/ unaware (4)

Q21 Considering the start date of your EPR system, how many years' worth of incident data have you been able to search for this survey? Ideally, up to 10 years. (e.g. 2014 - 2024)

________________________________________________________________

Q22 For reported DRUG ALLERGY incidents, what are the drugs involved, age group (≤17 or >17 years), and level of harm (no harm, low harm, moderate harm, severe harm or death), listing up to 10 cases prioritized by severity of harm, followed by the most recent incidents?
Please indicate the total cases below if more than 10 cases were reported.

Example: Case 1 (Amoxicillin, >17yo, low harm).

- Case 1 (allergen, age, level of harm) (1)
- Case 2 (allergen, age, level of harm) (2)
- Case 3 (allergen, age, level of harm) (3)
- Case 4 (allergen, age, level of harm) (4)
- Case 5 (allergen, age, level of harm) (5)
- Case 6 (allergen, age, level of harm) (7)
- Case 7 (allergen, age, level of harm) (8)
- Case 8 (allergen, age, level of harm) (9)
- Case 9 (allergen, age, level of harm) (10)
- Case 10 (allergen, age, level of harm) (11)
- If more than 10 cases are reported, please indicate the total number of cases below. (13)
- No drug allergy incidents reported (14)

Q23 For reported FOOD and OTHER NON-DRUG ALLERGY incidents, what are the allergens involved, age (confirm age via clinical record if required), reactions, if serious incident reported and level of harm (no harm, low harm, moderate harm, severe harm or death), listing up to 10 cases prioritized by severity of harm, followed by the most recent incidents? Please indicate the total cases below if more than 10 cases were reported. Example: Case 1 (Peanut, 3yo, anaphylaxis, serious incident reported, moderate harm).

- Case 1 (allergen, age, reaction, serious incident reported, level of harm) (1)
- Case 2 (allergen, age, reaction, serious incident reported, level of harm) (2)
- Case 3 (allergen, age, reaction, serious incident reported, level of harm) (3)
- Case 4 (allergen, age, reaction, serious incident reported, level of harm) (4)
- Case 5 (allergen, age, reaction, serious incident reported, level of harm) (5)
- Case 6 (allergen, age, reaction, serious incident reported, level of harm) (7)
- Case 7 (allergen, age, reaction, serious incident reported, level of harm) (8)
- Case 8 (allergen, age, reaction, serious incident reported, level of harm) (9)
- Case 9 (allergen, age, reaction, serious incident reported, level of harm) (10)
- Case 10 (allergen, age, reaction, serious incident reported, level of harm) (11)
- If more than 10 cases report, please indicate the total number of cases below. (13)
- No food allergy OR other non-drug allergy incidents reported (14)

Q24 For FOOD AND OTHER NON-DRUG ALLERGY incidents, how many of the incidents was the allergen clearly documented in patients notes/correspondence prior to the incident? Please insert the number of cases involved in each category. (e.g. 0 - 100)

- Food allergies documented correctly, please specify: (1) __________________________________________________
- Food allergies not documented, please specify: (2) __________________________________________________
- Non-drug allergies documented correctly, please specify: (3) __________________________________________________
- Non-drug allergies not documented, please specify: (4) __________________________________________________
- The food/ non-drug allergens were not previously known (7) __________________________________________________

Q25 For FOOD AND OTHER NON-DRUG ALLERGY incidents, how many of the incidents was the allergen correctly documented on the relevant field in EPR prior to incident (Cerner / Epic / Other)? Please insert the number of cases involved in each category. (e.g. 0 - 100)

- Food allergies documented correctly, please specify: (1) __________________________________________________
- Food allergies not documented, please specify: (2) __________________________________________________
- Non-drug allergies documented correctly, please specify: (3) __________________________________________________
- Non-drug allergies not documented, please specify: (4) __________________________________________________
- The food/ non-drug allergens were not previously known (5) __________________________________________________

Q26 What were the causes identified in the food or other non-drug incidents? (Multiple answers allowed)

- Allergy not recorded in EPR (1)
- Allergy recorded but not flagged/alerted (2)
- Staff did not check EPR (3)
- Incorrect substance administered due to similar names/packaging (4)
- System error or failure (5)
- Other (Please Specify) (6) __________________________________________________
- Unsure/ Don't know (7)

Q27 What challenges, if any, does your Trust face in accurately recording and managing allergy information in EPR systems?

________________________________________________________________

________________________________________________________________

Q28 What improvements do you suggest could be made at a national level to better manage allergy information in patient records?

________________________________________________________________

________________________________________________________________

Thank you very much for your input. This will provide the evidence and record for assisting in improvement of overall patients’ safety and quality of care.

**Other S3. BSACI Member Survey**

BSACI member Survey

Survey Flow

Block: Section 1: Demographic Information (8 Questions)

Standard: Section 2: Documentation of Non-Drug Allergies (6 Questions)

Standard: Section 3: Top Allergens Identification (2 Questions)

Standard: Section 4: Drug and Non-Drug Allergy Documentation (2 Questions)

Standard: Section 5: Allergy and Intolerance Documentation in EHR (4 Questions)

Standard: IF YES TO QUESTION 21, please complete the following questions. (Q22- 26) (5 Questions)

Standard: Section 6: Improving EPR Allergy Documentation (2 Questions)

Cover Statement- BSACI Survey on Allergy Recording in Electronic Patient Records (EPR) in NHS Trusts

INTRODUCTION Why are we asking for this information?

We are gathering information on how food, drug, and non-drug allergies are documented and managed within electronic patient records (EPR) across different NHS Trusts in the UK. This data will help identify current practices, challenges, and areas where national guidelines could be improved or introduced to improve patient safety and care. Your information is fundamental in helping us understand the variations in allergy documentation practices across NHS Trusts. By sharing your experiences and insights, you will contribute to potential improvements in allergy management and patient safety on a national level.

Who can take part?

BSACI members who are healthcare professionals (e.g. doctors, nurses, dietitians, pharmacist etc.) based in United Kingdom.

How long will it takes to complete this survey?

The survey should take approximately 10-15 minutes to complete. We appreciate your time and effort in providing valuable information.

How long will it takes to complete this survey?

The survey should take approximately 10-15 minutes to complete. We appreciate your time and effort in providing valuable information.

To decide if you would like to take part in this study, please read this participant information sheet. https://drive.google.com/file/d/1HNxQwMc5NOn6ojx4R855NO0BDrlLepqy/view?usp=share_link Please let us know if you are happy to take part in this survey:

- I confirm that I have read the information sheet dated 09/12/2024 (version 1.2) for the above study. I have had the opportunity to consider the information, ask questions and have had these answered satisfactorily. (9)
- I understand that my participation is voluntary and that I am free to withdraw at any time without giving any reason, without my legal rights being affected. (4)
- I understand that the survey is anonymous therefore my data cannot be withdrawn once I have submitted the survey. (5)
- I understand that the information collected about me will be used to support other research in the future, and may be shared anonymously with other researchers. (6)
- I understand that the survey responses maybe stored on Qualtrics EU data centre prior saving to Imperial College London cloud drive for analysis. (8)
- I agree to take part in the above study. (7)

Q1 Practice Location:

- North East and Yorkshire (1)
- North West (2)
- Midlands (3)
- East of England (4)
- London (5)
- South East (6)
- South West (7)
- Scotland (8)
- Wales (9)
- Northern Ireland (10)

Q2 Job description

- Doctor (Consultant) (1)
- Doctor (Non-consultant) (3)
- Doctor (GP) (2)
- Dentist (4)
- Nurse (allergy specialist) (5)
- Nurse (General) (6)
- Dietitian (7)
- Pharmacist (8)
- Scientist (9)
- Other (please specify) (10) __________________________________________________

Q3 Job Specialty (Select all if relevant)

- General Paediatrics (1)
- Internal Medicine (2)
- Allergy (3)
- Paediatric allergy (9)
- Immunology (7)
- Dermatology (4)
- Respiratory (5)
- Gastroenterology (6)
- Other (please specify) (8) __________________________________________________

Q4 Years of Experience in Allergy/Immunology:

|  | 0 | 5 | 10 | 15 | 20 | 25 | 30 | 35 | 40 | 45 | 50 |
| --- | --- | --- | --- | --- | --- | --- | --- | --- | --- | --- | --- |

| Years () | 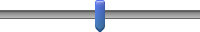 |
| --- | --- |

Q5 Practice type: (select all that relevant)

- Non-academic medical centre practice (7)
- Academic medical centre practice (1)
- Community hospital practice (2)
- Private practice with academic affiliation (3)
- Private allergy practice (4)
- Private multi-specialty practice (5)
- Other (please specify) (6) __________________________________________________

Q6 Does your institution have an Electronic Health Record (EHR) System?

- Yes (1)
- No (2)

Q7 Which company provides your EHR system?

- Cerner (1)
- Epic (2)
- System C (3)
- Dedalus (4)
- Altera (5)
- Other (please specify) (6) __________________________________________________

Q8 How frequently do you document non-drug allergies (e.g. food, environmental allergens, latex, dyes etc) in the EPR?

- Always (1)
- Most of the time (2)
- About half the time (3)
- Sometimes (4)
- Never (5)

Q9 What specific non-drug allergies do you most commonly document? (Select all that apply)

- Food allergies (1)
- Environmental allergies (e.g., pollen, dust mites) (2)
- Latex (3)
- Insect stings (4)
- Other (please specify) (5) __________________________________________________

Q10 Which entries do you review and/or update in the patient record? (Select all that apply)

- Drug allergies (1)
- Food allergies (2)
- Other non-drug allergies (e.g., environmental or contact allergens) (3)
- None of the above (4)

Q11 If you are documenting food allergies, how do you typically record food allergies in your documentation? (Select all that apply)

- Any IgE or non-IgE mediated food allergy (1)
- All IgE-mediated food allergies (2)
- Only selected IgE-mediated food allergies, please specify: (3) __________________________________________________

Q12 How would you rate the ease of documenting non-drug allergies in your current EPR system?

- Very Easy (1)
- Easy (2)
- Neutral (3)
- Difficult (4)
- Very Difficult (5)

Q13 Are there specific non-drug allergens that are difficult to code or document within your EPR system?

- Yes, please specify: (1) __________________________________________________
- No (2)

Q14 Are the 14 most common allergens (e.g., peanuts, shellfish, milk, etc.) easily and clearly identifiable in your EPR system?

- Yes (1)
- No, please describe the challenges you face: (2) __________________________________________________

Q15 What strategies do you use when the EPR system does not clearly identify or list a common allergen?

- Use of free-text entry (1)
- Use of generic code (2)
- Consultation with IT/Support (3)
- Other (please specify): (4) __________________________________________________

Q16 Does your EPR system allow you to document both a food allergy and a ‘no known drug allergy’ status for a patient?

- Yes (1)
- No, please specify how you handle this situation. (2) __________________________________________________

Q17 How do you handle cases where multiple allergies (both drug and non-drug) need to be documented? (Select all that relevant)

- Enter each allergy separately (1)
- Use of general terms and free-text (2)
- Prioritization of more severe allergies (3)
- Other (please specify): (4) __________________________________________________

Q18 What types of allergies can be entered into the allergy section of your electronic health record? (Check all that apply)

- Medication allergies (1)
- Medication intolerances (2)
- Food allergies (3)
- Food intolerances (e.g. lactose intolerance) (4)
- Environmental allergies (e.g. dust, mold) (5)
- Environmental intolerances (e.g. perfume) (6)
- Contact allergens (7)
- Latex allergy (8)
- Patient preferences (e.g. vegetarian, keto diet) (9)
- Other non-drug allergens, e.g. Chlorhexidine/ Povidone iodine/ Macrogol/ PEG-polyethylene glycol/ Polysorbate 20 or 80/ Mannitol (10) __________________________________________________

Q19 In your opinion, which allergies do you think should be entered into the electronic health record allergy section? (Check all that apply)

- Medication allergies (1)
- Medication intolerances (2)
- Food allergies (3)
- Food intolerances (e.g. lactose intolerance) (4)
- Environmental allergies (e.g. dust, mold) (5)
- Environmental intolerances (e.g. perfume) (6)
- Contact allergens (7)
- Latex allergy (8)
- Patient preferences (e.g. vegetarian, keto diet) (9)

Q20 Does your workplace have a guideline/SOP for the documentation of allergens on electronic patient records? (Select all that relevant)

- Yes- Drug allergy only (1)
- Yes- Drug and food allergy only (2)
- Yes- Drug, food and other non-drug allergy (3)
- No (4)

Q21 Are you aware of any patient safety incidents related to the administration of a food allergen or other non-drug allergen in your workplace?

- Yes (1)
- No (2)

Q22 How many cases of patient safety incidents related to administration of a food or non-drug allergens occurred or are you aware in your work place over the last 10 years?

- number of incidents- food allergen (1) __________________________________________________
- number of food allergy incidents reported via incident reporting system (2) __________________________________________________
- number of incidents- other non-drug allergen (3) __________________________________________________
- number of other non-drug allergy incidents reported via incident reporting system (4) __________________________________________________

Q23 Was practice changed after any of the incident investigations?

- Yes , please specify what was changed. (1) __________________________________________________
- No (2)
- Don't know (3)

Q24 Level of harm to patient and specify number of cases involved

- No Harm, please specify: (1) __________________________________________________
- Low Harm, please specify: (2) __________________________________________________
- Moderate Harm, please specify: (3) __________________________________________________
- Severe Harm, please specify: (4) __________________________________________________
- Death, please specify: (5) __________________________________________________
- Don't know, please specify if individual information. (7) __________________________________________________

Q25 What was the food involved in the allergic incidents, age and what were the reactions? (can insert "Don't know" in relevant section of text box if unsure, e.g. Peanut, Don't know, Urticaria)

- Food 1 (allergen, age, reaction) (1)
- Food 2 (allergen, age, reaction) (2)
- Food 3 (allergen, age, reaction) (3)
- Food 4 (allergen, age, reaction) (4)
- Food 5 (allergen, age, reaction) (5)
- Food 6 (allergen, age, reaction) (6)
- Food 7 (allergen, age, reaction) (7)
- Food 8 (allergen, age, reaction) (8)
- Food 9 (allergen, age, reaction) (9)
- Food 10 (allergen, age, reaction) (10)
- If more than 10 food incidents, please specify in box below (allergen, age, reaction) (11)
- No food involved incidents reported. (13)

Q26 What was the non-drug allergen involved in the allergic incidents, age and what were the reactions? (can insert "Don't know" in relevant section of text box if unsure, e.g. Peanut, Don't know, Urticaria)

- Non-drug 1 (allergen, age, reaction) (1)
- Non-drug 2 (allergen, age, reaction) (2)
- Non-drug 3 (allergen, age, reaction) (3)
- Non-drug 4 (allergen, age, reaction) (4)
- Non-drug 5 (allergen, age, reaction) (5)
- Non-drug 6 (allergen, age, reaction) (6)
- Non-drug 7 (allergen, age, reaction) (7)
- Non-drug 8 (allergen, age, reaction) (8)
- Non-drug 9 (allergen, age, reaction) (9)
- Non-drug 10 (allergen, age, reaction) (10)
- If more than 10 Non-drug incidents, please specify in box below (allergen, age, reaction) (11)
- No other non-drug allergy incident reported. (12)

Q27 What improvements would you suggest for the documentation of non-drug allergies in EPR systems? (Select all that relevant)

- Improved allergen databases on the EPR system (1)
- Better user interface for entering allergies (2)
- More comprehensive coding options (3)
- Integration of allergy history with clinical decision support (4)
- Other (please specify): (5) __________________________________________________

Q28 Would you be interested in having a national guidance or standard for the documentation of food and non-drug allergy recording in electronic patient records (EPR)?

- Yes (1)
- No (2)
